# Use of traditional medicine for hypertension, diabetes and hypercholesterolaemia measured in 71 surveys

**DOI:** 10.2471/BLT.25.293665

**Published:** 2025-09-29

**Authors:** Mubarak Ayodeji Sulola, Abla M Sibai, Albertino Damasceno, Alpamys Issanov, Antonio Sarria-Santamera, Binur Orazumbekova, Bolormaa Norov, Brice Bicaba, Corine Houehanou, David Guwatudde, Gibson B Kagaruki, Gladwell Gathecha, Jutta A. Jorgensen, Kibachio Joseph Mwangi, Kokou Agoudavi, Lela Sturua, Mary T Mayige, Mongal Gurung, Nahla Hwalla, Nuno Lunet, Omar Mwalim, Roy Wong McClure, Sarah Quesnel-Crooks, Silver Bahendeka, Rifat Atun, Till Bärnighausen, Justine Davies, David Flood, Pascal Geldsetzer, Lindsay Jaacks, Jennifer Manne-Goehler, Michaela Theilmann, Sebastian Vollmer, Maja E Marcus

**Affiliations:** a Department of Economics and Centre for Modern Indian Studies, University of Goettingen, Goettingen, Germany.; b Epidemiology and Population Health Department, American University of Beirut, Beirut, Lebanon.; c Faculty of Medicine, Eduardo Mondlane University, Maputo, Mozambique.; d School of Population and Public Health, University of British Columbia, Vancouver, Canada.; e Department of Biomedical Sciences, Nazarbayev University, Astana, Kazakhstan.; f Wolfson Institute of Population Health, Queen Mary University of London, London, England.; g National Center for Public Health, Ulaanbaatar, Mongolia.; h Institut National de Santé Publique, Ouagadougou, Burkina Faso.; i National School of Public Health, University of Parakou, Parakou, Benin.; j Department of Epidemiology and Biostatistics, Makerere University, Kampala, Uganda.; k National Institute for Medical Research, Dar es Salaam, United Republic of Tanzania.; l Division of Non-Communicable Diseases, Ministry of Health, Nairobi, Kenya.; m Department of Public Health, Copenhagen University, Copenhagen, Denmark.; n Togo Ministry of Health, Lomé, Togo.; o Non-Communicable Disease Department, National Center for Disease Control and Public Health, Tbilisi, Georgia.; p Health Research and Epidemiology Unit, Ministry of Health, Thimphu, Bhutan.; q Faculty of Agricultural and Food Sciences, American University of Beirut, Beirut, Lebanon.; r Department of Public Health and Forensic Sciences, and Medical Education, Faculty of Medicine, University of Porto, Porto, Portugal.; s Ministry of Health, Zanzibar City, United Republic of Tanzania.; t Office of Epidemiology and Surveillance, Costa Rican Social Security Fund, San José, Costa Rica.; u Caribbean Public Health Agency, Port of Spain, Trinidad and Tobago.; v Department of Internal Medicine, Uganda Martyrs University, Kampala, Uganda.; w Department of Global Health and Population, Harvard University, Boston, United States of America (USA).; x Heidelberg Institute of Global Health, Heidelberg University, Heidelberg, Germany.; y Department of Applied Health Research, University of Birmingham, Birmingham, England.; z Department of Medicine, University of Michigan, Ann Arbor, USA.; aa Division of Primary Care and Population Health, Stanford University, Stanford, USA.; ab Global Academy of Agriculture and Food Security, The University of Edinburgh, Midlothian, Scotland.; ac Division of Infectious Diseases, Brigham and Women's Hospital, 75 Francis Street, Boston, MA 02115, USA.

## Abstract

**Objective:**

To assess the pattern of traditional medicine use globally for treating hypertension, diabetes and hypercholesterolaemia.

**Methods:**

We pooled individual-level data from 309 745 non-pregnant people aged ≥ 15 years from 71 nationally representative surveys conducted in low- and middle-income countries between 2005 and 2021. We identified individuals with diagnosed hypertension, diabetes and hypercholesterolaemia who reported use of traditional medicine. For each condition, we estimated the prevalence of traditional medicine use at the global, regional and country-income level and the proportion using traditional medicine and biomedicine. We estimated the association between traditional medicine use and individual characteristics.

**Findings:**

The prevalence of traditional medicine use was 14.7% (95% confidence interval, CI: 12.7–16.9) for diabetes, 12.4% (95% CI: 10.0–15.3) for hypercholesterolaemia and 8.1% (95% CI: 7.3–9.0) for hypertension. Most individuals using traditional medicine for diabetes or hypercholesterolaemia also used biomedicine. Associations between sociodemographic characteristics and traditional medicine use varied between regions and health conditions. In the World Health Organization’s (WHO) Western Pacific Region, traditional medicine use for diabetes was significantly higher in males and younger adults, whereas use for hypertension was significantly higher in females and older adults. In the WHO African Region, traditional medicine use for diabetes and hypertension was higher in males and individuals with lower education.

**Conclusion:**

Our study shows a high prevalence of traditional medicine use for treating hypertension, diabetes and hypercholesterolaemia in low- and middle-income countries. Our results highlight the need to better understand the clinical interactions and risks of traditional medicine for improved cardiometabolic treatment.

## Résumé

**Objectif:**

Évaluer les habitudes d’utilisation de la médecine traditionnelle à l’échelle mondiale pour traiter l’hypertension, le diabète et l’hypercholestérolémie.

**Méthodes:**

Nous avons regroupé les données individuelles de 309 745 personnes non enceintes âgées de 15 ans ou plus. Ces données sont issues de 71 enquêtes représentatives à l’échelle nationale menées dans des pays à revenu faible ou intermédiaire entre 2005 et 2021. Nous avons identifié les personnes chez lesquelles une hypertension, un diabète ou une hypercholestérolémie avaient été diagnostiqués et qui déclaraient recourir à la médecine traditionnelle. Pour chaque affection, nous avons estimé la prévalence de l’utilisation de la médecine traditionnelle au niveau mondial, régional et national, ainsi que la proportion de personnes utilisant à la fois la médecine traditionnelle et la biomédecine. Nous avons estimé l’association entre l’utilisation de la médecine traditionnelle et les caractéristiques individuelles.

**Résultats:**

La prévalence du recours à la médecine traditionnelle était de 14,7% (intervalle de confiance (IC) à 95%: 12,7–16,9) pour le diabète, 12,4% (IC à 95%: 10,0–15,3) pour l’hypercholestérolémie et 8,1% (IC à 95%: 7,3–9,0) pour l’hypertension. La plupart des personnes utilisant la médecine traditionnelle pour le diabète ou l’hypercholestérolémie faisaient également appel à la biomédecine. Les associations entre les caractéristiques sociodémographiques et l’utilisation de la médecine traditionnelle variaient selon les régions et les problèmes de santé. Dans la région du Pacifique occidental de l’Organisation mondiale de la Santé (OMS), le recours à la médecine traditionnelle pour le diabète était significativement plus élevé chez les hommes et les jeunes adultes, tandis que son utilisation pour l’hypertension était significativement plus élevée chez les femmes et les personnes âgées. Dans la région Afrique de l’OMS, l’utilisation de la médecine traditionnelle pour le diabète et l’hypertension était plus élevée chez les hommes et les personnes ayant un niveau d’éducation moins élevé.

**Conclusion:**

Notre étude met en évidence une forte prévalence de l’utilisation de la médecine traditionnelle pour traiter l’hypertension, le diabète et l’hypercholestérolémie dans les pays à revenu faible et intermédiaire. Nos résultats soulignent la nécessité de mieux comprendre les interactions cliniques et les risques de la médecine traditionnelle afin d’améliorer le traitement cardiométabolique.

## Resumen

**Objetivo:**

Evaluar el patrón de uso de la medicina tradicional a nivel mundial para el tratamiento de la hipertensión, la diabetes y la hipercolesterolemia.

**Métodos:**

Se combinaron datos individuales de 309 745 personas no embarazadas de 15 años o más, procedentes de 71 encuestas nacionales representativas realizadas en países de ingresos bajos y medios entre 2005 y 2021. Se identificaron las personas con diagnóstico de hipertensión, diabetes e hipercolesterolemia que declararon utilizar medicina tradicional. Para cada afección, se estimó la prevalencia del uso de medicina tradicional a nivel mundial, regional y según el nivel de ingresos del país, así como el porcentaje de personas que usaban simultáneamente medicina tradicional y biomedicina. Además, se estimó la asociación entre el uso de medicina tradicional y las características individuales.

**Resultados:**

La prevalencia del uso de medicina tradicional fue del 14,7% (intervalo de confianza, IC, del 95%: 12,7-16,9) para la diabetes; del 12,4% (IC del 95%: 10,0-15,3) para la hipercolesterolemia; y del 8,1% (IC del 95%: 7,3-9,0) para la hipertensión. La mayoría de las personas que utilizaban medicina tradicional para la diabetes o la hipercolesterolemia también usaban simultáneamente biomedicina. Las asociaciones entre las características sociodemográficas y el uso de medicina tradicional variaron según la región y la afección. En la Región del Pacífico Occidental de la Organización Mundial de la Salud (OMS), el uso de medicina tradicional para la diabetes fue significativamente mayor en hombres y adultos jóvenes, mientras que su uso para la hipertensión fue mayor en mujeres y personas mayores. En la Región de África de la OMS, el uso de medicina tradicional para la diabetes y la hipertensión fue más elevado en hombres y en personas con menor nivel educativo.

**Conclusión:**

Este estudio muestra una elevada prevalencia del uso de la medicina tradicional para el tratamiento de la hipertensión, la diabetes y la hipercolesterolemia en los países de ingresos bajos y medios. Los resultados destacan la necesidad de comprender mejor las interacciones clínicas y los riesgos asociados al uso de la medicina tradicional para optimizar el tratamiento de las enfermedades cardiometabólicas.

## ملخص

**الغرض:**

تقييم نمط استخدام الطب التقليدي عالميًا لعلاج ارتفاع ضغط الدم، والسكري، وفرط كوليسترول الدم.

**الطريقة:**

قمنا بجمع بيانات فردية من 309745 امرأة غير حامل، تتراوح أعمارهن بين 15 عامًا فأكثر، من 71 استبيانًا تمثيليًا على المستوى الوطني، تم إجراؤها في الدول ذات الدخل المنخفض والمتوسط بين عامي 2005 و2021. قمنا بتحديد الأفراد الذين تم تشخيص إصابتهم بارتفاع ضغط الدم والسكري وفرط كوليسترول الدم، والذين أبلغوا عن استخدامهم للطب التقليدي. لكل حالة، قمنا بتقدير معدل انتشار استخدام الطب التقليدي على المستوى العالمي والإقليمي ومستوى دخل الدولة، ونسبة من يستخدمون الطب التقليدي والطب البيولوجي معًا. كما قمنا بتقدير العلاقة بين استخدام الطب التقليدي والخصائص الفردية.

**النتائج:**

بلغ معدل انتشار استخدام الطب التقليدي %14.7 (بفاصل ثقة %95: 12.7 إلى 16.9) لمرض السكري، و%12.4 (بفاصل ثقة %95: 10.0 إلى 15.3) لفرط كوليسترول الدم، و%8.1 (بفاصل ثقة %95: 7.3 إلى 9.0) لارتفاع ضغط الدم. إن معظم الأفراد الذين يستخدمون الطب التقليدي لعلاج السكري أو ارتفاع كوليسترول الدم، قد استخدموا أيضًا الطب البيولوجي معه. وتباينت الارتباطات بين الخصائص الاجتماعية والديموغرافية واستخدام الطب التقليدي باختلاف المناطق والظروف الصحية. ففي منطقة غرب المحيط الهادئ لمنظمة الصحة العالمية، كان استخدام الطب التقليدي لعلاج السكري أعلى بشكل ملحوظ لدى الذكور والبالغين الأصغر سناً، بينما كان استخدامه لعلاج ارتفاع ضغط الدم أعلى بشكل ملحوظ لدى الإناث وكبار السن. وفي المنطقة الأفريقية لمنظمة الصحة العالمية، كان استخدام الطب التقليدي لعلاج السكري وارتفاع ضغط الدم أعلى لدى الذكور والأفراد ذوي التعليم المنخفض.

**الاستنتاج:**

تُظهر الدراسة لدينا ارتفاعًا لمعدل انتشار استخدام الطب التقليدي لعلاج ارتفاع ضغط الدم والسكري وارتفاع كوليسترول الدم في الدول ذات الدخل المنخفض والدخل المتوسط. تسلط النتائج لدينا الضوء على الحاجة إلى فهم أفضل للتفاعلات السريرية ومخاطر الطب التقليدي لتحسين علاج أمراض القلب والأوعية الدموية.

## 摘要

**目的:**

旨在评估全球范围内使用传统医学治疗高血压、糖尿病和高胆固醇血症的相关情况。

**方法:**

我们汇集了 2005 年至 2021 年期间在中低收入国家开展的 71 项具有全国代表性的调查所涉及 309,745 名年龄在 15 岁及以上的非妊娠人士的个人数据。我们确定了那些已确诊患有高血压、糖尿病和高胆固醇血症且报告其使用过传统医学疗法的个人。针对每一种疾病，我们分别从全球、区域和国家收入层面估算了传统医学的使用率以及同时使用传统医学和生物医学的比例。我们评估了传统医学使用情况与个人特征之间的关联性。

**结果:**

使用传统医学治疗糖尿病、高胆固醇血症和高血压的比例分别为 14.7%（95% 置信区间 (CI)：12.7–16.9）、12.4%（95% CI：10.0–15.3）和 8.1%（95% CI：7.3–9.0）。大多数人在使用传统医学治疗糖尿病或高胆固醇血症时还会同时使用生物医学。社会人口学特征与传统医学使用情况之间的关联性因区域和健康状况的不同而存在差异。在世卫组织 (WHO) 西太平洋区域，患有糖尿病的男性和年轻成年人使用传统医学的比例明显更高，而患有高血压的女性和老年人使用传统医学的比例明显更高。在世卫组织非洲区域，患有糖尿病和高血压的男性和受教育程度较低的人员使用传统医学的比例更高。

**结论:**

我们的研究表明：在中低收入国家，人们使用传统医学治疗高血压、糖尿病和高胆固醇血症的比例较高。我们的研究结果显著表明：我们需要更深入地了解传统医学的临床相互作用和潜在风险，从而改善其对心血管代谢疾病的疗效**。**

## Резюме

**Цель:**

Оценить картину использования в мире традиционной медицины для лечения гипертензии, диабета и гиперхолестеринемии.

**Методы:**

Авторы объединили индивидуальные данные по 309 745 небеременным людям в возрасте ≥ 15 лет, полученные в ходе 71 национально-репрезентативного опроса, проведенного в странах с низким и средним уровнем дохода населения в период между 2005 и 2021 годами. Были выявлены опрошенные с диагностированной гипертензией, диабетом и гиперхолестеринемией, которые сообщали об использовании традиционной медицины. Для каждого заболевания была дана оценка частоты использования традиционной медицины на мировом, региональном и государственном (с учетом дохода) уровнях, а также было определено соотношение между использованием традиционной и биомедицины. Авторы оценили наличие связи между индивидуальными характеристиками пациентов и использованием традиционной медицины.

**Результаты:**

Частота использования традиционной медицины составила 14,7% (95%-й доверительный интервал, ДИ: 12,7–16,9) для диабета, 12,4% (95%-й ДИ: 10,0–15,3) для гиперхолестеринемии и 8,1% (95%-й ДИ: 7,3–9,0) для гипертензии. Большинство опрошенных, которые принимали традиционные средства против диабета и гиперхолестеринемии, также лечились и методами биомедицины. Связь между социодемографическими характеристиками и использованием традиционной медицины варьировалась в зависимости от регионов и заболеваний. В Западно-Тихоокеанском регионе Всемирной организации здравоохранения (ВОЗ) использование традиционной медицины при диабете было существенно выше у мужчин и молодых людей, тогда как использование при гипертензии было существенно выше у женщин и людей старшего возраста. В Африканском регионе ВОЗ использование традиционной медицины при диабете и гипертензии было выше у мужчин и лиц с более низким уровнем образования.

**Вывод:**

Исследование показало высокую частоту обращения к традиционной медицине при лечении гипертензии, диабета и гиперхолестеринемии в странах с низким и средним уровнем дохода. Результаты подчеркивают необходимость лучшего понимания клинических взаимодействий и рисков традиционной медицины для улучшения лечения кардиометаболических заболеваний.

## Introduction

Despite the rising burden of noncommunicable diseases due to hypertension, diabetes and hypercholesterolaemia in low- and middle-income countries,^1^^–^^3^ large gaps in care for these conditions persist.^4^^–^^6^ Earlier studies suggest that unmet needs in the conventional care system, including high costs of medications, inaccessibility of health care, misconceptions about cardiovascular diseases,^7^^,^^8^ and strong cultural preferences may contribute to the use of traditional medicine as a source of care in people with cardiometabolic conditions.^7^^–^^13^

Traditional medicine is defined by the World Health Organization (WHO) as “codified or non-codified systems for health care and well-being comprising practices, skills, knowledge and philosophies originating in different historical, cultural contexts, that are distinct from and pre-date biomedicine, evolving scientifically for current use from an experience-based origin.”^14^ Conceptually, traditional medicine ranges from provider-directed services, such as medicinal plants formulations, Ayurvedic treatment and spiritual healing, to self-directed practices, such as meditation, acupressure, prayer and use of music.^15^ Safe and effective traditional medicine can help advance the goal of universal health coverage by: improving trust and the use of conventional health care, particularly in societies where traditional medicine is considered culturally appropriate;^7^^,^^16^ contributing to pharmaceutical drug discovery;^17^^,^^18^ and reducing health-care costs through, for example, fewer hospital stays and medication prescriptions.^19^^,^^20^ Given these contributions and the widespread use of traditional medicine, WHO developed a traditional medicine strategy that aims to both harness the potential contributions of traditional medicine to health and promote the safe and effective use of traditional medicine.^21^ While 98 WHO Member States report having national policies on traditional medicine, challenges around regulation, health-system integration, safety and quality, and training of traditional medicine providers persist.^21^^,^^22^

To address these challenges, evidence on patterns of traditional medicine use and health service use is important. The reported prevalence of traditional medicine use is estimated to be between 8.8% and 68.0% for hypertension treatment,^10^^,^^23^^–^^25^ and between 28.9% and 89.0% for diabetes.^12^^,^^26^^–^^32^ Information on traditional medicine use for hypercholesterolaemia or the use of traditional medicine with other health services is scarce. Evidence specific to these cardiometabolic risk factors from nationally representative samples is rare.^32^^,^^33^ We therefore aimed to: (i) estimate the prevalence of traditional medicine use for the treatment of hypertension, diabetes and hypercholesterolaemia using nationally representative data from 71 surveys; (ii) show how traditional medicine is used with other health services; and (iii) describe individual-level characteristics associated with traditional medicine use by health condition and geographical region.

## Methods

### Study design and participants

Our cross-sectional study used individual-level health survey data obtained through the Global Health and Population Project on Access to Care for Cardiometabolic Diseases. This process identified all surveys matching the following inclusion criteria: (i) nationally representative and conducted from 2005 onwards in low- and middle-income countries; (ii) conducted at the individual level; (iii) with a response rate greater than 50%; and (iv) with blood glucose, blood pressure and/or blood cholesterol data.^34^ Further details on the processes used are summarized in the online repository.^35^ Based on these criteria, we identified 71 surveys from 70 countries and territories (Box 1; we included two surveys from United Republic of Tanzania, one survey representing mainland and one Zanzibar) with data on self-reported traditional medicine use (online repository).^35^

Box 1 **Countries and territories by WHO region included in the study on the use of traditional medicine for hypertension, diabetes and hypercholesterolaemia**

**African Region**

Algeria, Benin, Botswana, Burkina Faso, Cabo Verde, Comoros, Eritrea, Eswatini, Ethiopia, Gambia, Kenya, Lesotho, Liberia, Malawi, Mozambique, Namibia, Rwanda, Sao Tome and Principe, Sierra Leone, Togo, Uganda, United Republic of Tanzania, Zambia, Zanzibar

**Region of the Americas**

Belize, Bolivia (Plurinational State of), Costa Rica, Ecuador, Grenada, Guyana, Paraguay, Saint Lucia

**South-East Asia Region**

Bhutan, Myanmar, Sri Lanka, Timor-Leste

**European Region**

Azerbaijan, Belarus, Georgia, Kazakhstan, Kyrgyzstan, Republic of Moldova, Tajikistan, Turkmenistan, Ukraine

**Eastern Mediterranean Region**

Afghanistan, Iraq, Jordan, Lebanon, Libya, Morocco, West Bank and Gaza Strip, Sudan

**Western Pacific Region**

Cambodia, China, Fiji, Indonesia, Kiribati, Lao People’s Democratic Republic, Marshall Islands, Mongolia, Nauru, Palau, Samoa, Solomon Islands, Tokelau, Tonga, Tuvalu, Vanuatu, Viet Nam, Wallis and Futuna

Of these 71 surveys, 66 were surveys within the WHO STEPwise approach to NCD risk factor surveillance (STEPS); see online repository for the data available in these surveys and for country-specific sampling procedures.^35^ Since not all country surveys reported data on traditional medicine use for each health condition, we created separate samples for hypertension, diabetes and hypercholesterolaemia (online repository).^35^ For each health condition, our eligible sample included all non-pregnant individuals with no missing biomarkers, self-reported diagnosis of the health condition, self-reported use of traditional medicine, age and sex.

### Outcomes

To define our analysis sample, we first identified all individuals with hypertension, diabetes or hypercholesterolaemia. Of these people, we included only respondents diagnosed with the health condition (online repository).^35^ We defined hypertension as systolic blood pressure ≥ 140 mmHg or diastolic blood pressure ≥ 90 mmHg or reporting the use of medication for hypertension.^4^ We defined diabetes as haemoglobin A1C (HbA1c) reading ≥ 6.5%, fasting plasma glucose ≥ 7.0 mmol/L, random plasma glucose ≥ 11.1 mmol/L, or reporting the use of glucose-lowering medication.^5^ We defined hypercholesterolaemia as a cholesterol reading of ≥ 6.2 mmol/L or use of lipid-lowering medications.^6^ We considered a respondent had been diagnosed if they reported that a doctor or other health worker had ever told them that they had diabetes, hypertension or hypercholesterolaemia.

In our analysis, traditional medicine use refers to self-reported current use of medicinal plants or traditional remedies for the health conditions (online repository).^35^ Although interpretations might vary across cultures,^15^ biologically-based remedies, including medicinal plants, are the most commonly used traditional medicine, followed by faith-based remedies such as prayers and spiritual guidance, and mind–body methods such as massage and traditional bone-setting.^33^ Our three main variables of interest were: currently taking any medicinal plants or traditional remedy for the treatment of raised blood pressure, diabetes or hypercholesterolaemia. We created a secondary set of binary variables showing whether respondents used traditional medicine only, both used traditional medicine and biomedicine, or used biomedicine only. Biomedicine was any medication prescribed by a doctor or health worker, such as statins or any other medication.^36^ In a supplementary analysis, we included self-reported consultation with a traditional healer for any of the three health conditions (online repository).^35^ Lastly, we included self-reported age, sex and education.

### Statistical analysis

We estimated the overall proportions of respondents with single, co- or multimorbid diagnoses who self-reported use of traditional medicine for the treatment of hypertension, diabetes and/or hypercholesterolaemia, overall and by country. We estimated the proportion of our sample that reported the use of traditional medicine only or the use of traditional medicine with biomedicine overall, by WHO regions and by World Bank income groups (online repository).^35^ We also estimated the proportion of diagnosed respondents who used traditional medicine and self-reported ever visiting a traditional healer. For each health condition, we assessed the association between traditional medicine use and sex, age, education, use of biomedicine and country using a modified Poisson regression model^37^ (online repository).^35^

We used Stata, version 16.1 (StataCorp. LP, College Station, United States of America) for all analyses. We rescaled sampling weights according to the 2015 population of each country.^38^ In cases of missing survey weights, the country averages were assigned, and a complete case analysis was used for other data (online repository).^35^ We conducted some sensitivity checks. We reported estimates for the subsample of countries that had data available on traditional medicine use for all three health conditions, and the subsample of surveys from the past 10 years only.

## Results

### Sample characteristics

All individuals with hypertension, diabetes or hypercholesterolaemia are shown in the online repository.^35^ About half self-reported a diagnosis by a doctor or health worker. Overall, 41 637 individuals from 71 surveys had diagnosed hypertension, 10 041 from 64 surveys had diagnosed diabetes and 5696 from 46 surveys had diagnosed hypercholesterolaemia. Women made up between 55.0% (95% confidence interval, CI: 50.4–59.4) and 58.0% (95% CI: 56.6–59.4) of the samples. The sample characteristics of diagnosed individuals are available in the online data repository.^35^

### Traditional medicine use

**Fig. 1:**
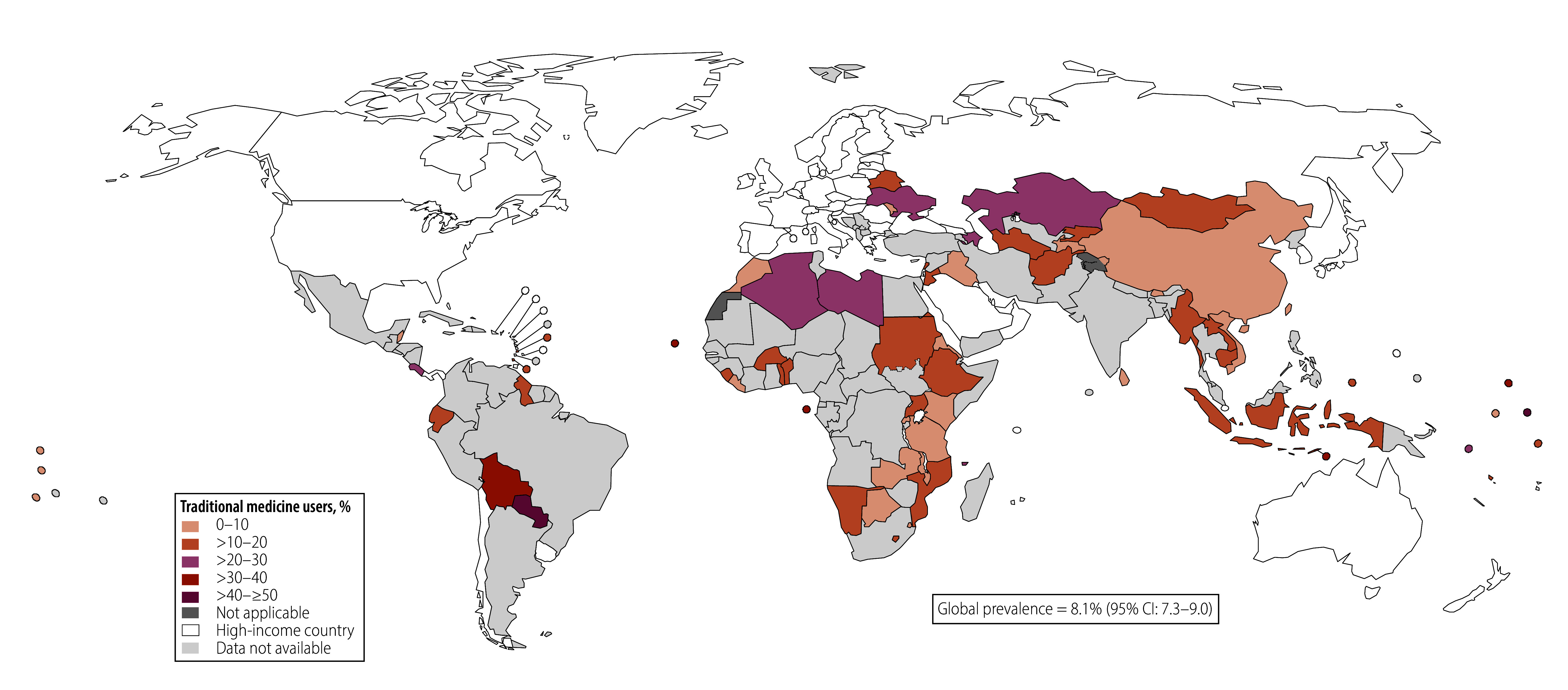
Proportion of respondents with diagnosed hypertension using traditional medicine, by country

**Fig. 2:**
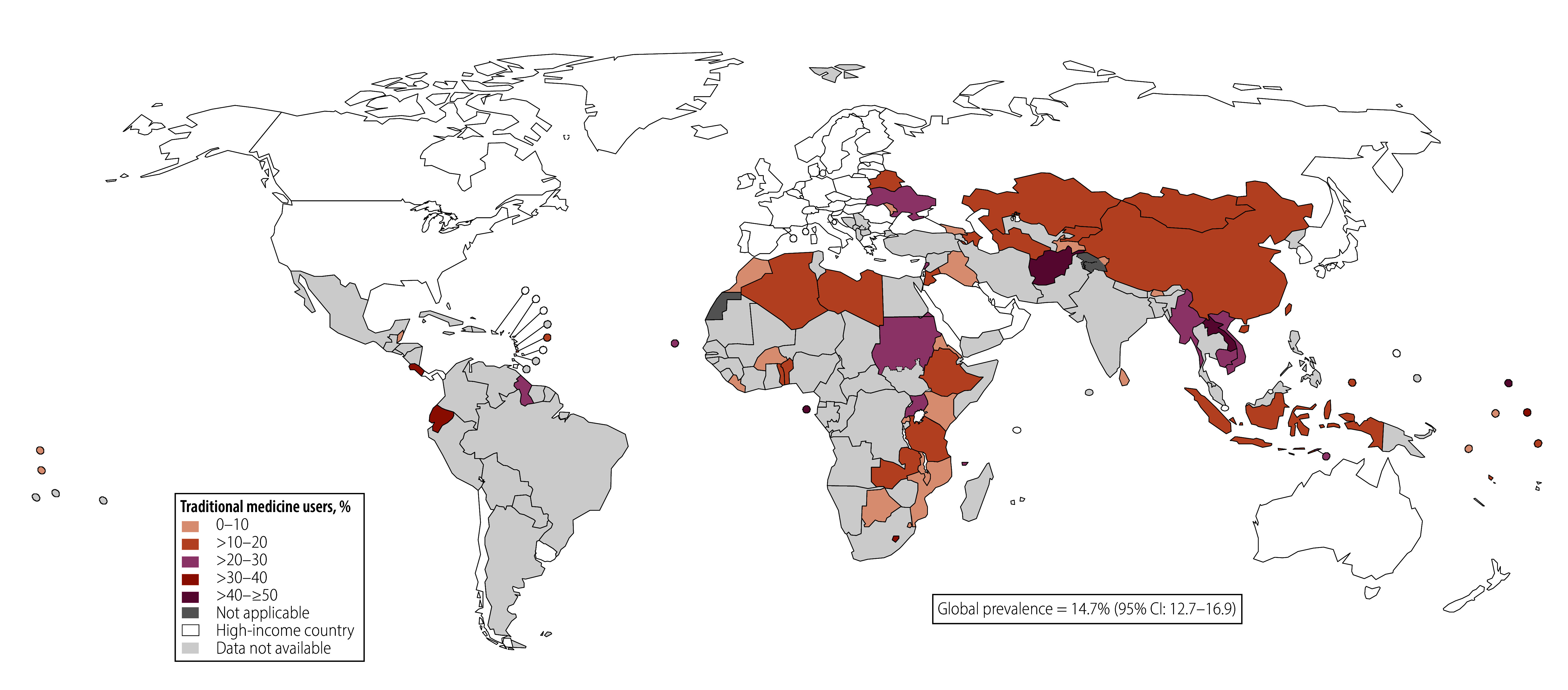
Proportion of respondents with diagnosed diabetes using traditional medicine, by country

**Fig. 3:**
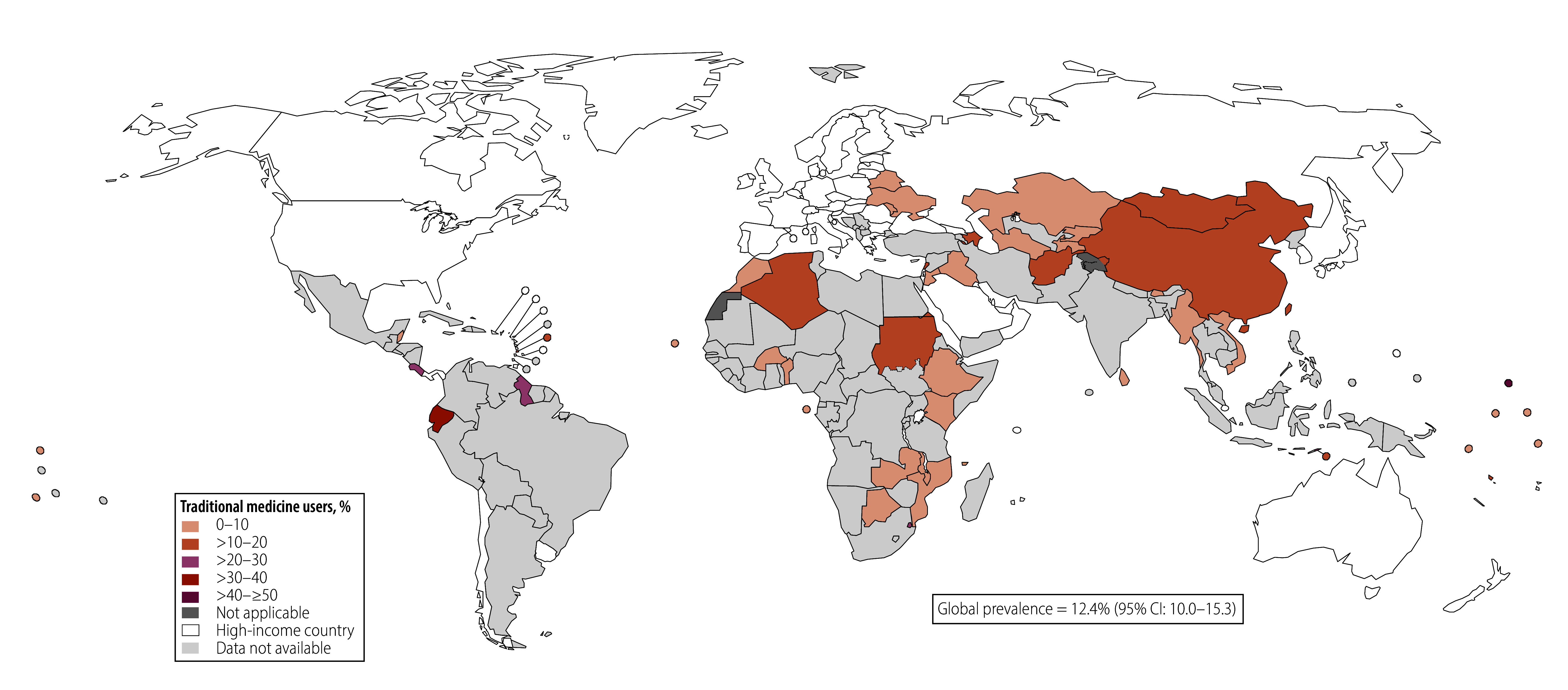
Proportion of respondents with diagnosed hypercholesterolaemia using traditional medicine, by country

As shown in Fig. 1, Fig. 2 and Fig. 3 the overall use of traditional medicine was highest for the treatment of diabetes (14.7%; 95% CI: 12.7–16.9), followed by hypercholesterolaemia (12.4%; 95% CI: 10.0–15.3) and then hypertension (8.1%; 95% CI: 7.3–9.0). This ranking persisted even with the sample of countries that had data on all three conditions (online repository).^35^ The timing of the surveys did not alter this pattern (online repository).^35^ When restricting the analysis to individuals with any two or all three conditions, we found a similar ranking of traditional medicine use across health conditions. Traditional medicine use was most prevalent for treatment of diabetes, except in the sample with both diabetes and hypercholesterolaemia, where traditional medicine use for treating hypercholesterolaemia was more prevalent (Table 1).

CI: confidence interval.

Notes: estimates account for survey design and weighting by each country’s population size in 2015. The enlarged circles represent small island countries.

CI: confidence interval.

Notes: estimates account for survey design and weighting by each country’s population size in 2015. The enlarged circles represent small island countries.

CI: confidence interval.

Notes: estimates account for survey design and weighting by each country’s population size in 2015. The enlarged circles represent small island countries.

**Table 1:** Proportion of respondents using traditional medicine, by number of health conditions

Group	Use of traditional medicine, % (95% CI)		
	Hypertension	Diabetes	Hypercholesterolaemia
**Multimorbidity^a^**	5.09 (3.35–7.67)	15.29 (9.98–22.72)	8.15 (5.29–12.35)
**Co-morbidity^b^**			
Hypertension and diabetes	7.69 (5.52–10.61)	16.36 (11.82–22.21)	NA
Hypertension and hypercholesterolaemia	6.04 (4.25–8.52)	NA	9.41 (6.67–13.11)
Diabetes and hypercholesterolaemia	NA	16.22 (8.36–29.13)	18.15 (9.22–32.64)

CI: confidence interval; NA: not applicable.

^a^ These individuals self-reported diagnosis of all three health conditions.

^b^ These individuals self-reported diagnosis of any two of the health conditions.

Note: All estimates account for complex survey design and are weighted by each country’s population in 2015 and are estimated in the subsample of more than 45 countries with data for each relevant health condition.

We found large heterogeneity in traditional medicine use across countries (online repository).^35^ The proportion of individuals using traditional medicine for hypertension ranged from 1.0% (95% CI: 0.4–2.5) in Kenya to 45.4% (95% CI: 40.8–50.1) in Paraguay. For diabetes, it ranged from < 1% in Burkina Faso, Liberia, Mozambique and Rwanda to 50.5% (95% CI: 35.1–65.8) in Sao Tome and Principe; and for hypercholesterolaemia, it ranged from < 1% in nine countries to 100% in Malawi.

### Use with other health services

**Fig. 4:**
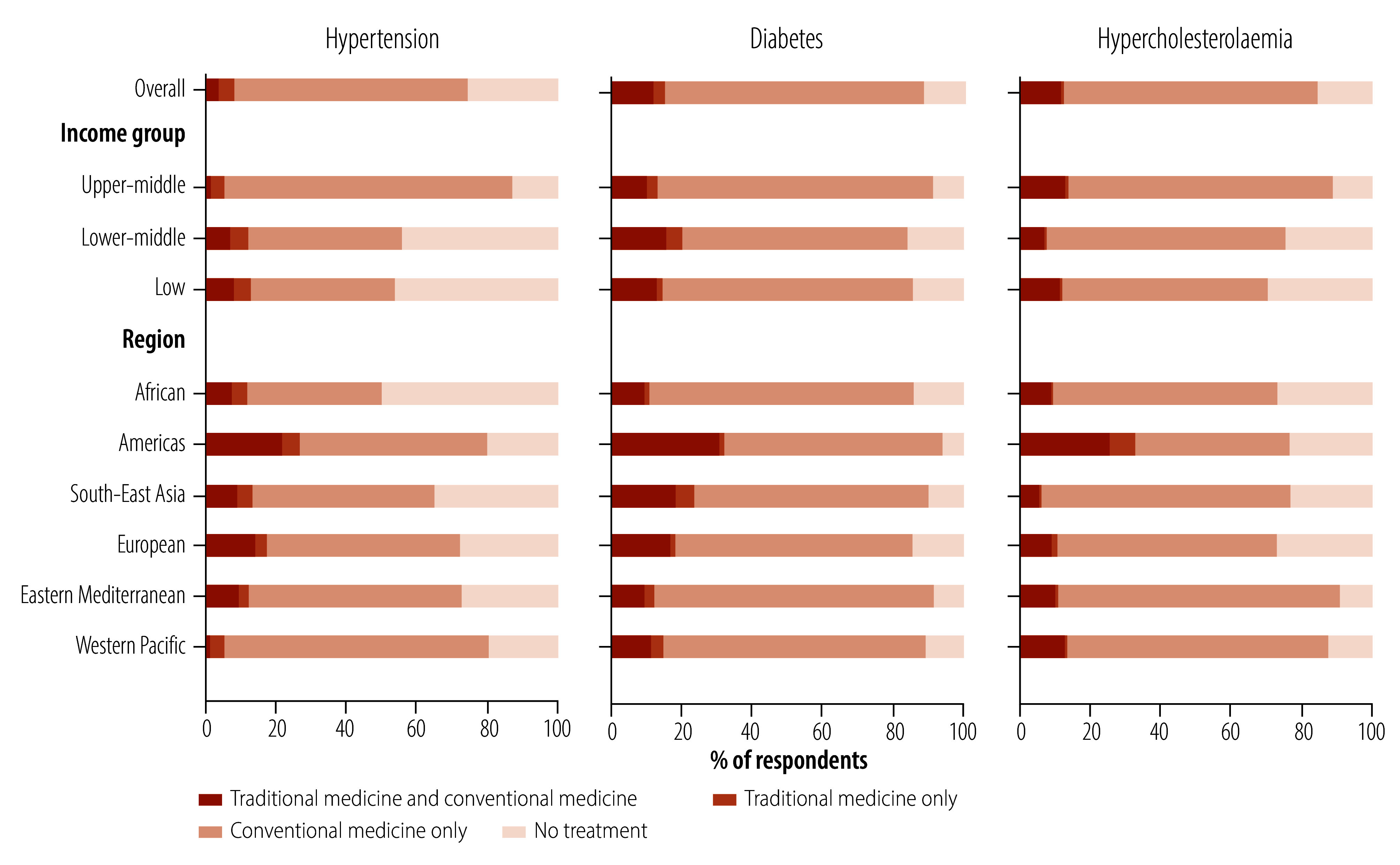
Proportion of respondents with diagnosed hypertension, diabetes and hypercholesterolaemia using traditional medicine with or without biomedicine, by country income group and WHO region

Notes: estimates account for complex survey design and are weighted by each country’s population in 2015. The income group are based on the World Bank Income group at the time of the survey. The denominator is all individuals with health conditions who have self-reported diagnoses.

Fig. 4 shows the pattern of the use of traditional medicine and biomedicine by region and World Bank income group (online repository).^35^ Use of traditional medicine only for treatment of hypertension was greater than both use of traditional medicine and biomedicine. Specifically, 4.4% (95% CI: 3.7–5.2) used traditional medicine only, while 3.8% (95% CI: 3.3–4.3) used both traditional and biomedicine. For treatment of diabetes and hypercholesterolaemia, use of both was greater (more than 10% of individuals) than use of traditional medicine only: 3.2% (95% CI: 2.3–4.4) for diabetes and 0.7% (95% CI: 0.3–1.3) for hypercholesterolaemia.

By World Bank income groups, use of traditional medicine for hypertension and diabetes (with and without biomedicine) was greater in low-income countries and lower-middle-income countries than upper-middle-income countries: hypertension 12.7% (95% CI: 9.9–16.3) and 11.9% (95% CI: 10.8–13.0) versus 5.5% (95% CI: 4.5–6.7); and diabetes 14.0% (95% CI: 9.6–20.0) and 20.0% (95% CI: 16.1–24.4) versus 12.6% (95% CI: 10.2–15.4). For treating hypercholesterolaemia, the use of traditional medicine (with and without biomedicine) was highest in upper-middle-income countries. Similar to the overall proportions, use of both traditional and biomedicine was higher than the use of traditional medicine alone across the income groups for both diabetes and hypercholesterolaemia. For hypertension, the use of traditional medicine alone was more prevalent than the use of both only in upper-middle income countries.

By WHO region, use of both traditional and biomedicine was greater than use of traditional medicine alone in most regions (Fig. 4). Only for the treatment of hypertension in the Western Pacific Region, the use of traditional medicine alone was greater than the use of both. The South-East Asia Region had the highest use of traditional medicine alone for the treatment of diabetes. The Region of the Americas had the highest use of traditional medicine only for the treatment of hypertension, and for hypercholesterolaemia, about nine times the global average estimate: The Regions of the Americas 7.1% (95% CI: 4.9–10.2) and worldwide 0.7% (95% CI: 0.4–1.4).

About half of individuals using traditional medicine for treatment of hypertension and diabetes, and about a third using traditional medicine for treatment of hypercholesterolaemia, self-reported ever visiting a traditional healer for their condition (online repository).^35^

### Factors associated with use

Using traditional medicine for diabetes was significantly and negatively associated with being female, older than 35 years, having a primary-school education, compared with no formal education and using biomedicine. Using traditional medicine was also significantly negatively associated with the use of biomedicine for hypertension. However, using traditional medicine was significantly associated with use of biomedicine for hypercholesterolaemia (online repository).^35^ Disaggregation by geographical region showed substantial heterogeneity in these associations.

Associations of traditional medicine use with sociodemographic characteristics substantially varied between health conditions and by regional groups (Table 2). For treating hypertension in the Western Pacific Region, use of traditional medicine was significantly associated with being female (risk ratio, RR: 1.52; 95% CI: 1.09–2.12) and older than 34 years (e.g. ≥ 55 years, RR: 2.90; 95% CI: 1.78–4.72). Traditional medicine use was negatively associated with using biomedicine (RR: 0.08; 95% CI: 0.04–0.14). In the African Region, traditional medicine use for hypertension was negatively associated with being female (RR: 0.66; 95% CI: 0.45–0.98) and having secondary school education or higher (RR: 0.70; 95% CI: 0.50–0.99) compared with no formal education, and was positively associated with using biomedicine (RR: 1.72; 95% CI: 1.12–2.65). For diabetes in Africa, using traditional medicine was also negatively associated with being female (RR: 0.27; 95% CI: 0.15–0.48) and having at least secondary school education (RR: 0.40; 95% CI: 0.19–0.87) compared with no formal education. In the Western Pacific Region, traditional medicine use for diabetes was also negatively associated with being female (RR: 0.68; 95% CI: 0.46–1.00), being older than 34 years (e.g. ≥ 55 years, RR: 0.39; 95% CI: 0.20–0.78), having a primary school education compared with no formal education (RR: 0.55; 95% CI: 0.36–0.86) and using biomedicine (RR: 0.50; 95% CI: 0.33–0.75). Living in a rural area was only positively associated with traditional medicine use for diabetes in Europe, but negatively associated with traditional medicine use for hypertension in the South-East Asia Region (online repository).^35^ We restricted the regression models to countries that had data on all three conditions (online repository).^35^

**Table 2:** Association between traditional medicine use and sociodemographic characteristics for hypertension and diabetes, by WHO region

Condition	RR (95% CI)					
	African Region	Region of the Americas	South-East Asia Region	European Region	Eastern Mediterranean Region	Western Pacific Region
**Hypertension**						
Sex						
Male	Ref	Ref	Ref	Ref	Ref	Ref
Female	0.66 (0.45–0.98)	0.99 (0.82–1.19)	1.29 (0.99–1.69)	1.27 (1.00–1.61)	0.92 (0.67–1.27)	1.52 (1.09–2.12)
Age group, in years						
15–34	Ref	Ref	Ref	Ref	Ref	Ref
35–44	0.62 (0.28–1.39)	1.37 (0.80–2.37)	0.93 (0.46–1.85)	1.25 (0.67–2.35)	1.12 (0.67–1.89)	1.84 (0.99–3.44)
45–54	0.82 (0.37–1.82)	1.74 (1.06–2.84)	1.04 (0.54–2.01)	1.35 (0.76–2.39)	1.15 (0.69–1.92)	3.24 (1.89–5.54)
≥ 55	0.79 (0.33–1.92)	1.83 (1.13–2.99)	1.43 (0.70–2.92)	1.77 (0.97–3.22)	0.96 (0.58–1.58)	2.90 (1.78–4.72)
Education						
No formal schooling	Ref	Ref	Ref	Ref	Ref	Ref
Primary schooling	0.80 (0.50–1.27)	0.93 (0.70–1.24)	1.16 (0.77–1.75)	0.40 (0.17–0.97)	0.76 (0.54–1.06)	0.90 (0.68–1.21)
Secondary school or above	0.70 (0.50–0.99)	0.89 (0.65–1.22)	0.91 (0.73–1.14)	0.47 (0.21–1.07)	1.05 (0.73–1.51)	1.31 (0.87–1.95)
Biomedicine use						
No	Ref	Ref	Ref	Ref	Ref	Ref
Yes	1.72 (1.12–2.65)	1.53 (1.22–1.92)	1.41 (1.08–1.85)	1.54 (1.07–2.24)	1.41 (1.04–1.92)	0.08 (0.04–0.14)
**Diabetes**						
Sex						
Male	Ref	Ref	Ref	Ref	Ref	Ref
Female	0.27 (0.15–0.48)	1.32 (0.86–2.02)	1.67 (1.10–2.54)	0.83 (0.51–1.34)	0.84 (0.59–1.19)	0.68 (0.46–1.00)
Age group, in years						
15–34	Ref	Ref	Ref	Ref	Ref	Ref
35–44	0.91 (0.20–4.19)	0.26 (0.09–0.81)	2.98 (0.80–11.04)	0.53 (0.16–1.73)	0.67 (0.37–1.21)	0.20 (0.05–0.76)
45–54	1.10 (0.26–4.72)	0.50 (0.19–1.32)	1.61 (0.37–7.01)	0.45 (0.16–1.27)	0.57 (0.29–1.11)	0.25 (0.10–0.61)
≥ 55	1.65 (0.43–6.29)	0.45 (0.18–1.14)	2.30 (0.52–10.13)	0.50 (0.21–1.16)	0.45 (0.27–0.75)	0.39 (0.20–0.78)
Education						
No formal schooling	Ref	Ref	Ref	Ref	Ref	Ref
Primary schooling	0.62 (0.25–1.52)	1.60 (0.74–3.44)	1.11 (0.58–2.11)	0.33 (0.04–2.61)	0.71 (0.47–1.06)	0.55 (0.36–0.86)
Secondary school or above	0.40 (0.19–0.87)	1.00 (0.43–2.31)	1.30 (0.77–2.21)	1.35 (0.20–9.28)	0.77 (0.52–1.15)	0.72 (0.43–1.21)
Biomedicine use						
No	Ref	Ref	Ref	Ref	Ref	Ref
Yes	1.09 (0.40–3.01)	1.89 (0.74–4.83)	0.63 (0.27–1.48)	2.34 (0.99–5.49)	1.69 (0.91–3.13)	0.50 (0.33–0.75)

CI: confidence interval; Ref: reference category; RR: risk ratio.

Note: The analysis accounts for survey sampling weights, weighting countries according to their population and controlling for country-fixed effects. We excluded Tokelau in the Western Pacific Region because the survey did not include information on education level.^35^

## Discussion

Using data from 71 nationally representative surveys, we found that traditional medicine use for the treatment of diabetes, hypercholesterolaemia and hypertension was estimated at 14.7%, 12.4%, and 8.1%, respectively. There was substantial variation across regions. Use of both traditional and biomedicine was more prevalent for treating diabetes and hypercholesterolaemia, relative to hypertension. The association between traditional medicine use and individual-level characteristics differed substantially between regions and between health conditions within regions. Our study provides nationally representative and geographic-wide evidence about patterns of traditional medicine use and its use with other health services for the treatment of key cardiometabolic risk factors, representing a total population of more than 2 billion people. Our study provides useful data for: (i) health workers given the relevance of the use of traditional and biomedicine; (ii) research on underlying causes of traditional medicine use given regional- and disease-specific patterns of traditional medicine use; and (iii) policy-making on the integration of safe and effective traditional medicine use into existing care structures for cardiometabolic conditions.^21^

Our findings align with the more conservative estimates of traditional medicine use in low- and middle-income countries reported in previous studies, which range from 8.8% to 68.0% for hypertension^10^^,^^23^^–^^25^ and from 28.9% to 89.0% for diabetes.^12^^,^^26^^–^^32^ Many of the studies with higher estimates were based on non-nationally representative samples and were primarily conducted in health facilities.^31^^–^^33^ These factors may overestimate traditional medicine use due to selection bias of individuals with higher treatment-seeking behaviour. In contrast, our estimates rely on nationally representative survey sampling with cross-country comparability, including survey design, recall period and measure of traditional medicine use. Studies that also use nationally representative sampling more closely align with our findings.^39^ National and cross-country studies on the prevalence of traditional medicine use have reported rates ranging between < 2% and 19% in China, Ghana, India, Mexico, the Philippines, the Russian Federation and South Africa.^23^^,^^40^^,^^41^ Taken together, these figures suggest that the use of traditional medicine may be less common than often assumed, although not negligible, especially for diabetes. Consequently, acknowledging and integrating evidence-based traditional medicine remedies may help bridge care gaps for these conditions through mediums such as timely referral of patients to conventional care practice. Such programmes are already being tested for other health conditions, including human immunodeficiency virus (HIV) infection.^16^ In our study, the large heterogeneity of traditional medicine use across regions and countries implies that condition-specific strategies on traditional medicine use are necessary, especially given the uniqueness of traditional medicine remedies and practices across regions and countries.^21^

We found substantial use of both traditional and biomedicine, indicating that people do not necessarily opt out of conventional medications when taking traditional remedies. Given the potential adverse health outcomes associated with unaligned use of traditional medicine for cardiovascular conditions,^10^^,^^27^ also reported for other health issues such as HIV^16^ and mental disorders,^42^ our findings suggest that safe and evidence-based integration is imperative. Such integration is important to mitigate adverse health outcomes since: (i) up to 70% of traditional medicine users reportedly do not disclose their use to conventional health workers;^29^^,^^32^ and (ii) use of traditional medicine is reported to be a significant predictor of non-adherence to treatment prescribed by conventional practitioners.^10^^,^^43^ Additionally, we also found non-negligible rates of traditional medicine use without biomedicine, especially in the Western Pacific Region, where over 3 in 4 traditional medicine users for hypertension do so without biomedicine. Countries and territories in this region have indigenous traditional medicine, which is used at both individual and primary health care levels.^44^ These countries, especially in the Oceania area, are also reported to face structural health-care issues and lower coverage of conventional diabetes care than other regions.^45^^,^^46^ The use of traditional medicine only highlights the need for integrated health systems, given safety regulations, potential harms and unproven efficacy of many remedies.^21^ As a wide variety of traditional medicine exists across countries, the potential risks can differ by remedy and include adverse side-effects, lack of efficacy and harmful interactions with biomedicine.^47^

The likelihood of using traditional medicine with or without biomedicine differed across health conditions and regions. Differences by health condition could be related to lifestyle- and diet-related perceptions of hypercholesterolaemia, which places traditional remedies as acceptable complements to conventional drugs. People may use traditional medicine to reinforce conventional treatment or as part of broader dietary or wellness practices, especially where lipid management is relatively less emphasized in public health programmes. Our finding on the low use of traditional medicine and visiting a traditional healer aligns with previous studies that found most remedies were either sourced from social networks^13^^,^^29^^,^^48^ or self-prepared.^4^ Commonly cited reasons for not visiting a traditional healer among traditional medicine users include concerns about the credibility of traditional healer services on issues such as dosing, the expertise of healers, remedy regulations and witchcraft.^12^^,^^13^^,^^49^ Determining whether specific remedies are safe complements to, or substitutes for, biomedicine requires context-specific investigation. This information is crucial for traditional medicine users, particularly where dosing is inconsistent and safety regulations are limited.^13^^,^^21^

Our findings on traditional medicine users in Africa align with previous research in the region, which showed that traditional medicine users for hypertension and diabetes were more likely to be male^10^^,^^25^ and people of lower socioeconomic status.^25^^,^^48^ In contrast, we found women to be more likely to use traditional medicine for hypertension in South-East Asia, European and Western Pacific Regions, which aligns with findings from previous research in China,^41^ India,^50^ Sri Lanka^28^ and also higher-income countries.^9^^,^^40^ Furthermore, previous findings show age to be associated with traditional medicine use in Africa.^25^^,^^27^^,^^29^^,^^48^ While we found no such association, we found that younger individuals in the Americas, the Eastern Mediterranean and Western Pacific Regions were more likely to use traditional medicine for diabetes, while younger individuals in the European Region were more likely to use traditional medicine for hypercholesterolaemia. Several factors might contribute to this finding, including easier accessibility to traditional medicine remedies,^12^ affordability^9^^,^^10^ and barriers to accessing conventional health care. Previous research has reported that poorer conventional health-care delivery contributes to traditional medicine use,^13^ and younger individuals are less likely to receive conventional treatment for hypertension, and to be treated and controlled for diabetes.^4^^,^^5^

A limitation of our study is that the interpretation of medicinal plants or traditional remedies can vary individually, especially in the absence of details of the many remedies being used in the survey questionnaires. Thus, respondents might under- or over-report use of remedies depending on what they consider a traditional remedy to be.^15^ Additionally, self-reporting the use of traditional medicine is subject to social desirability bias and can differ in magnitude by region and individual groups. For instance, respondents may have under-reported their use of traditional medicine to field staff if they associated them with biomedicine practitioners, since physical and biomarker measurements were collected in the surveys. Likewise, excluding individuals with missing or false reporting on traditional medicine use could have resulted in some selection bias, although this share of individuals was minimal.

In conclusion, our findings highlight the need for more research to better understand potential clinical interactions, risks and safety issues associated with traditional medicine use for cardiometabolic health conditions. Policies to ensure safe and evidence-based traditional medicine remedies for individuals with these conditions should be a global priority.
